# Trehalose Attenuates In Vitro Neurotoxicity of 6-Hydroxydopamine by Reducing Oxidative Stress and Activation of MAPK/AMPK Signaling Pathways

**DOI:** 10.3390/ijms251910659

**Published:** 2024-10-03

**Authors:** Danijela Stevanovic, Ljubica Vucicevic, Maja Misirkic-Marjanovic, Tamara Martinovic, Milos Mandic, Ljubica Harhaji-Trajkovic, Vladimir Trajkovic

**Affiliations:** 1 Institute of Microbiology and Immunology, Faculty of Medicine, University of Belgrade, Dr. Subotica 1, 11000 Belgrade, Serbia; danijela.stevanovic@med.bg.ac.rs (D.S.);; 2 Institute for Biological Research “Sinisa Stankovic”—National Institute of Republic of Serbia, University of Belgrade, Bulevar Despota Stefana 142, 11000 Belgrade, Serbia; maja.misirkic@ibiss.bg.ac.rs (M.M.-M.);

**Keywords:** trehalose, Parkinson’s disease, 6-hydroxydopamine, MPP^+^, JNK, p38 MAPK, AMP-activated protein kinase, oxidative stress, mitochondrial damage

## Abstract

The effects of trehalose, an autophagy-inducing disaccharide with neuroprotective properties, on the neurotoxicity of parkinsonian mimetics 6-hydroxydopamine (6-OHDA) and 1-methyl-4-phenylpiridinium (MPP^+^) are poorly understood. In our study, trehalose suppressed 6-OHDA-induced caspase-3/PARP1 cleavage (detected by immunoblotting), apoptotic DNA fragmentation/phosphatidylserine externalization, oxidative stress, mitochondrial depolarization (flow cytometry), and mitochondrial damage (electron microscopy) in SH-SY5Y neuroblastoma cells. The protection was not mediated by autophagy, autophagic receptor p62, or antioxidant enzymes superoxide dismutase and catalase. Trehalose suppressed 6-OHDA-induced activation of c-Jun N-terminal kinase (JNK), p38 mitogen-activated protein kinase (MAPK), and AMP-activated protein kinase (AMPK), as revealed by immunoblotting. Pharmacological/genetic inhibition of JNK, p38 MAPK, or AMPK mimicked the trehalose-mediated cytoprotection. Trehalose did not affect the extracellular signal-regulated kinase (ERK) and mechanistic target of rapamycin complex 1 (mTORC1)/4EBP1 pathways, while it reduced the prosurvival mTORC2/AKT signaling. Finally, trehalose enhanced oxidative stress, mitochondrial damage, and apoptosis without decreasing JNK, p38 MAPK, AMPK, or AKT activation in SH-SY5Y cells exposed to MPP^+^. In conclusion, trehalose protects SH-SY5Y cells from 6-OHDA-induced oxidative stress, mitochondrial damage, and apoptosis through autophagy/p62-independent inhibition of JNK, p38 MAPK, and AMPK. The opposite effects of trehalose on the neurotoxicity of 6-OHDA and MPP+ suggest caution in its potential development as a neuroprotective agent.

## 1. Introduction

Parkinson’s disease (PD) is a progressive neurodegenerative disorder with environmental/genetic etiology, characterized by alpha-synuclein aggregation, oxidative stress, mitochondrial dysfunction, and death of nigrostriatal dopaminergic neurons [1]. Parkinsonian mimetics 6-hydroxy-dopamine (6-OHDA) and 1-methyl-4-phenylpyridini- um (MPP^+^), a neurotoxic metabolite of 1-methyl-4-phenyl-1,2,3,6-tetrahydropyridine (MPTP), are widely used in experimental models of PD [2]. Monoamine oxidases (MAO) catalyze the oxidation of 6-OHDA to neurotoxic metabolites and conversion of MPTP to MPP^+^, which induce oxidative stress, mitochondrial damage, and mainly apoptotic death in dopaminergic neurons [3]. The lysosomal degradation of oxidized/misfolded proteins and damaged mitochondria through macroautophagy (hereafter autophagy) is a plausible therapeutic strategy for combating neurodegeneration in PD [4]. However, excessive autophagy can be neurotoxic [5], and 6-OHDA and MPTP/MPP^+^ induce cytoprotective autophagy, cytotoxic autophagy, or cytotoxic autophagy blockade in a context-dependent manner [6]. Neurotoxicity of 6-OHDA and MPP^+^ is associated with the activation of the intracellular energy sensor adenosine monophosphate-activated protein kinase (AMPK) and mitogen-activated protein kinase (MAPK) family members p38, extracellular signal-regulated kinase (ERK), and c-Jun N-terminal kinase (JNK) [7,8,9]. The two enzyme complexes containing the mechanistic target of rapamycin (mTOR), called mTOR complex 1 (mTORC1) and mTORC2, also regulate neuronal function and survival by phosphorylating their respective targets, including the eukaryotic translation initiation factor 4E-binding protein 1 (4EBP1) and anti-apoptotic protein kinase B/AKT [10].

Trehalose is a non-reducing glucose disaccharide found in insects, plants, fungi, and bacteria, which stabilizes macromolecules/membranes and protects cells from various stresses [11]. Trehalose reduces oxidative stress, mitochondrial dysfunction, neuroinflammation, and neuronal death in experimental models of neurodegeneration, mainly by degrading neurotoxic protein aggregates via AMPK- and transcription factor EB-dependent autophagy [12,13]. Trehalose prevents the neurotoxicity of H_2_O_2_ or proteasome inhibitors through suppression of AMPK or ERK, respectively [14,15], and modulation of AKT, p38 MAPK, or JNK accompanies the protective action of trehalose in non-neurodegenerative disease models [16,17,18]. The effects of trehalose on 6-OHDA/MPP^+^ neurotoxicity are insufficiently explored, with conflicting results in different experimental settings. Trehalose-mediated suppression of 6-OHDA neurotoxicity in rats was associated with the increase in antioxidant enzyme activity, antioxidant transcription factor Nrf2, and the expression of autophagy marker microtubule-associated protein 1 light-chain 3 (LC3)-II and autophagic cargo receptor p62 [19]. However, the causal relationships between the neuroprotective/antioxidant activity of trehalose and the effects on autophagy/p62 were not assessed. While trehalose protected mice from MPTP-mediated neuronal damage [20,21], it increased the in vitro toxicity of MPP^+^ in SK-N-SH neuroblastoma cells via an unknown autophagy-independent mechanism [22,23].

Given the conflicting results and the lack of mechanistic evidence, we investigated the effects of trehalose on 6-OHDA and MPP^+^ neurotoxicity in vitro, focusing on the molecular mechanisms. We show that trehalose protects SH-SY5Y human neuroblastoma cells from 6-OHDA- but not MPP^+^-triggered apoptosis through autophagy/p62-indepen- dent reduction of oxidative stress, mitochondrial damage, and the activity of JNK/p38 MAPK/AMPK signaling pathways.

## 2. Results

### 2.1. Trehalose Inhibits 6-OHDA Toxicity towards SH-SY5Y Cells

**Figure 1:**
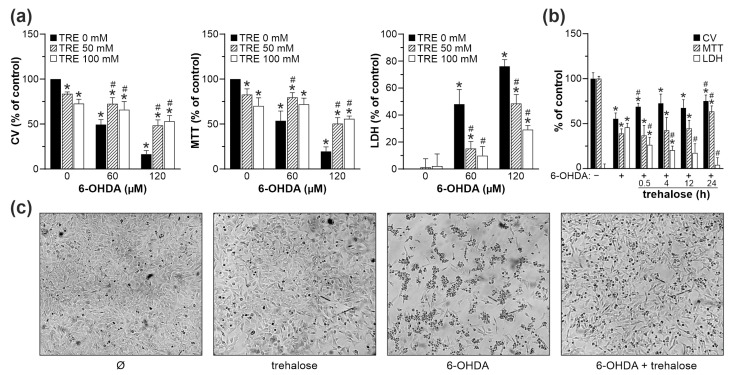
Trehalose inhibits 6-OHDA toxicity in SH-SY5Y cells. (**a**–**c**) SH-SY5Y cells were incubated with or without 50 mM (**a**) or 100 mM (**a**–**c**) of trehalose (TRE) for 24 h (**a**,**c**) or the indicated times (**b**), and then treated or not with 60 µM (**a**–**c**) or 120 µM (**a**) of 6-OHDA for another 24 h. (**a**) Cell viability was determined by crystal violet (CV) and MTT assays, and the cytotoxicity was assessed by LDH release (**a**,**b**). The data are mean ± SD values from three independent experiments (**a**) or mean ± SD values of triplicate measurements from a representative of two experiments (**b**) (* *p* < 0.05 vs. no treatment; # *p* < 0.05 vs. 6-OHDA). Cell shape and numbers were examined by light microscopy (40×), and the representative light micrographs from three independent experiments are shown (**c**).

**Figure 2:**
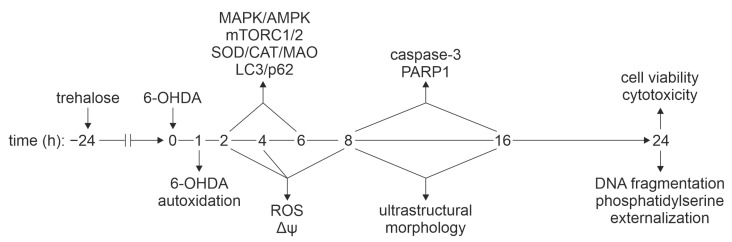
Experimental design for exploring the mechanisms of trehalose-mediated cytoprotection. After 24 h preincubation with trehalose (100 mM), SH-SY5Y cells were treated with 6-OHDA (60 µM). The autoxidation of 6-OHDA was measured in a cell-free system. MAPK/AMPK/mTORC1/2 signaling, autophagy (LC3, p62), oxidative stress/antioxidant defense (6-OHDA autoxidation, intracellular ROS, SOD, CAT, MAO), mitochondrial membrane potential (Δψ), autophagic/apoptotic ultrastructural morphology (autophagic vesicles, mitochondrial/nuclear morphology), apoptotic markers (caspase-3, PARP1, phosphatidylserine externalization, DNA fragmentation), and cell viability/cytotoxicity were assessed at the indicated time points. CAT, catalase; PARP1, poly (ADP-ribose) polymerase 1; ROS, reactive oxygen species; SOD, superoxide dismutase.

We first determined the maximum nontoxic dose of trehalose by exposing SH-SY5Y neuroblastoma cells to various concentrations (50–200 mM) of the compound for 24 and 48 h. Trehalose exhibited a dose- and time-dependent reduction in the number of adherent cells and mitochondrial dehydrogenase activity, as assessed by crystal violet and 3-(4,5-dimethylthiazol-2-yl)-2,5-diphenyltetrazolium bromide (MTT) assays, respectively (Table 1). However, only the highest trehalose concentration (200 mM) significantly increased cell death, as determined by the release of lactate dehydrogenase (LDH), a marker of cell membrane damage (Table 1). This indicates that the decrease in crystal violet and MTT values in cells exposed to 50 mM and 100 mM trehalose was primarily due to an antiproliferative effect. This was confirmed by cell cycle analysis in cells stained with DNA-intercalating dye propidium iodide, showing a G_0_/G_1_ block but no apoptotic DNA fragmentation (sub-G_0_/G_1_ compartment) upon the treatment with 50 or 100 mM of trehalose for 48 h (Supplementary Figure S1). Therefore, these doses were considered non-toxic and used to examine the ability of trehalose to protect SH-SY5Y cells from 6-OHDA. To that aim, SH-SY5Y cells were incubated for 24 h with 6-OHDA (60 µM and 120 µM) in the presence or absence of trehalose (50 mM and 100 mM), which was added to the cell culture 24 h before neurotoxin treatment. Although trehalose alone slightly decreased cell proliferation, it significantly increased cell number (crystal violet) and mitochondrial respiration (MTT), and reduced cell death (LDH release) in 6-OHDA-treated cell cultures (Figure 1a). The trehalose concentration of 100 mM was chosen for further experiments because it was more efficient than a 50 mM dose in preventing cell death, as confirmed by the LDH assay (Figure 1a). This was not apparent in crystal violet and MTT assays, since the ability of 100 mM trehalose to suppress 6-OHDA toxicity was probably partly masked by its antiproliferative effect (Figure 1a). By varying the preincubation time with trehalose, we also demonstrated that the longest preincubation time of 24 h provided the best protection against 6-OHDA (Figure 1b). In accordance with the observed protection, light microscopy has shown that trehalose prevented the decrease in cell number, as well as cell rounding and detachment from the plate surface observed in 6-OHDA-treated cultures (Figure 1c). Based on the above findings, we designed an experimental protocol to test the effects of the 24 h preincubation with 100 mM trehalose on apoptosis, oxidative stress, antioxidant defense, mitochondrial health, autophagy, and MAPK/AMPK/mTORC1/2 signaling in SH-SY5Y cells exposed to 60 µM of 6-OHDA (Figure 2). The time points for all analyses were defined based on the literature data and our previous/preliminary findings.

### 2.2. Trehalose Inhibits 6-OHDA-Induced Caspase Activation and Apoptosis in SH-SY5Y Cells

**Figure 3:**
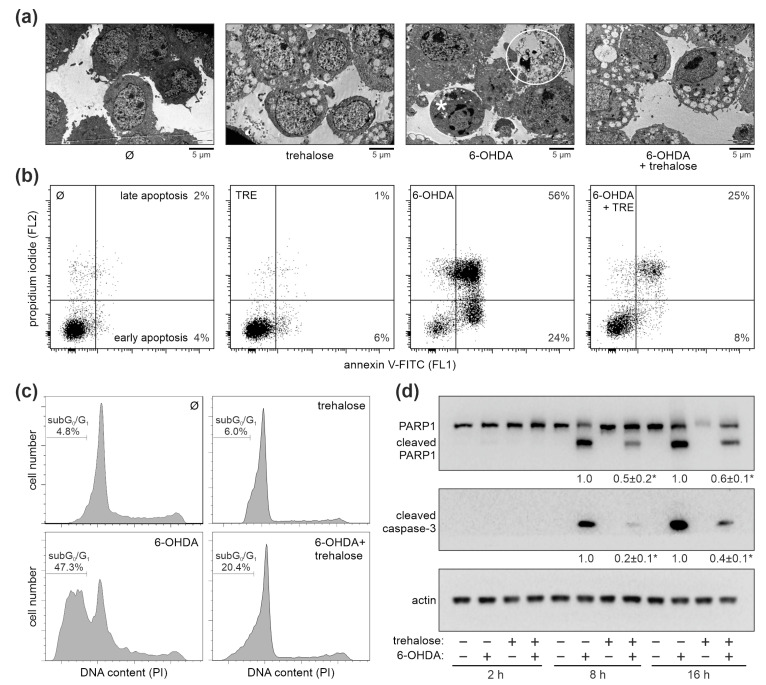
Trehalose inhibits 6-OHDA-induced apoptosis of SH-SY5Y cells. (**a**–**d**) SH-SY5Y cells were incubated with or without trehalose (100 mM) for 24 h and then treated or not with 6-OHDA (60 µM) for another 16 h (**a**), 24 h (**b**,**c**), or the indicated times (**d**). TEM visualization of the ultrastructural morphology shows an apoptotic cell with condensed chromatin but intact cell membrane (white asterisk) and a cell with membrane damage undergoing secondary necrosis (white circle) in 6-OHDA-, but not 6-OHDA + trehalose-treated cells (**a**). Phosphatidylserine externalization (**b**) and DNA fragmentation (**c**) were assessed by flow cytometric analysis of annexin/PI and PI-stained cells, respectively. PARP1 cleavage and caspase-3 activation were analyzed by immunoblotting, with densitometry values below the relevant bands (mean ± SD, n = 3; * *p* < 0.05 vs. 6-OHDA) (**d**). The representative TEM images (**a**), flow cytometry histograms (**b**,**c**), and immunoblots (**d**) from two (**a**–**c**) or three (**d**) independent experiments are shown.

We next examined the effects of trehalose on 6-OHDA-induced apoptotic events in SH-SY5Y cells. Ultrastructural analysis by transmission electron microscopy (TEM) revealed a cytoplasmic vacuolization in cells exposed to trehalose alone but without any signs of cell damage or death. On the other hand, trehalose markedly attenuated 6-OHDA-induced morphological changes characteristic of apoptosis, including nuclear chromatin condensation (pyknosis) and fragmentation (karyorrhexis) (Figure 3a). Accordingly, flow cytometry analysis revealed that trehalose diminished the 6-OHDA-induced increase in the proportion of apoptotic annexin V^+^ cells with phosphatidylserine externalized to the outer leaflet of the plasma membrane (Figure 3b). Moreover, flow cytometry analysis of the sub-G_0_/G_1_ (hypodiploid) compartment of the cell cycle in propidium iodide-stained cells confirmed that trehalose markedly reduced 6-OHDA-induced DNA fragmentation (Figure 3c). Finally, the immunoblot analysis demonstrated a reduced activation of the apoptosis-executing caspase-3 and the subsequent cleavage of its substrate, the DNA-repairing enzyme poly (ADP-ribose) polymerase 1 (PARP1) in 6-OHDA- treated cells that were preincubated with trehalose (Figure 3d). Taken together, these results show that trehalose inhibits 6-OHDA-induced apoptotic death of SH-SY5Y cells.

### 2.3. Trehalose Prevents 6-OHDA-Induced Oxidative Stress and Mitochondrial Damage in SH-SY5Y Cells

**Figure 4:**
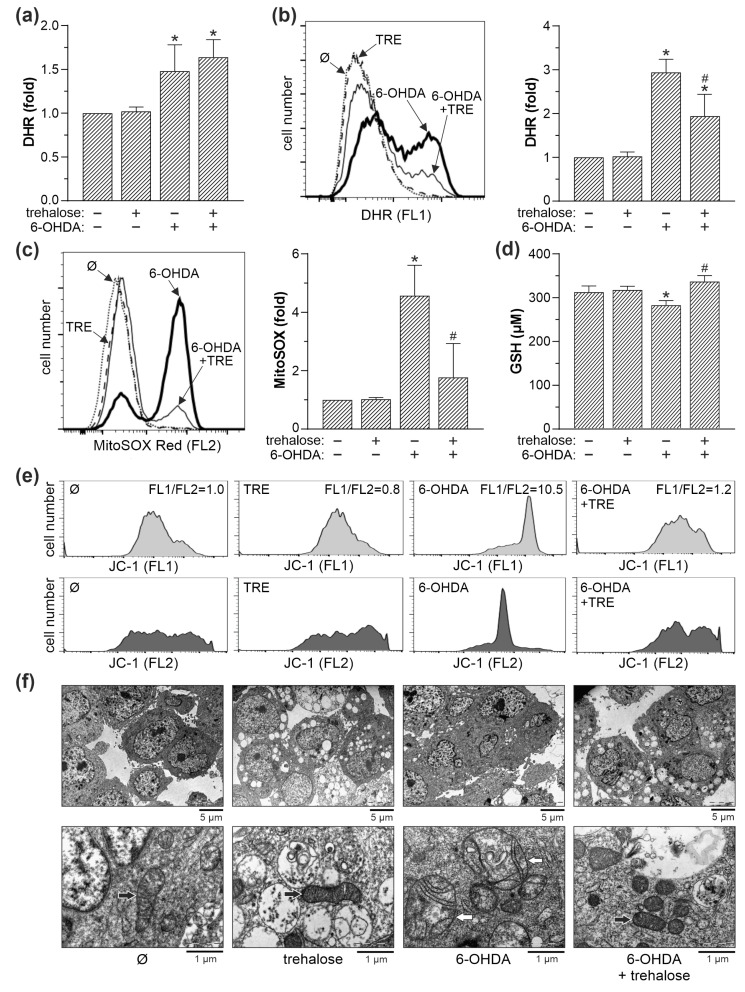
Trehalose prevents 6-OHDA-induced oxidative stress and mitochondrial damage in SH-SY5Y cells. (**a**) Cell-free complete cell culture medium with or without trehalose (100 mM) was incubated in cell-culturing conditions for 24 h. Then, 6-OHDA (60 µM) was added for the next 1 h, and ROS levels were determined by measuring DHR fluorescence. (**b**–**f**) SH-SY5Y cells were incubated with or without trehalose (TRE; 100 mM) for 24 h and then exposed or not to 6-OHDA (60 µM) for another 8 h. Intracellular ROS production (**b**), mitochondrial superoxide production (**c**), and mitochondrial depolarization (**e**) were determined by flow cytometric analysis of DHR-, MitoSOX- and JC-1-stained cells, respectively. Total reduced glutathione (GSH) was determined colorimetrically (**d**), while mitochondrial morphology was analyzed by TEM (black and white arrows showing normal and damaged mitochondria, respectively) (**f**). The data are mean ± SD values from three (**a**,**c**) or four (**b**) independent experiments, or mean ± SD values of triplicate measurements from a representative of two experiments (**d**) (* *p* < 0.05 vs. no treatment; # *p* < 0.05 vs. 6-OHDA). The representative histograms (**b**,**c**,**e**) or electron micrographs (**f**) are shown.

Since oxidative stress is the main mediator of 6-OHDA-induced neurotoxicity, we assessed the effect of trehalose on 6-OHDA-triggered production of reactive oxygen species (ROS). Trehalose failed to reduce the fluorescence of a redox-sensitive dye dihydrorhodamine (DHR) in cell-free conditions (Figure 4a), indicating that its cytoprotective effect is not due to the direct neutralization of ROS. On the other hand, trehalose suppressed 6-OHDA-induced production of total ROS and mitochondrial superoxide in SH-SY5Y cells, as confirmed by flow cytometry analysis of cells stained with DHR and MitoSOX, respectively (Figure 4b,c). The spectrophotometry analysis demonstrated that the levels of reduced glutathione, which is consumed during oxidative stress, were decreased by 6-OHDA and restored in trehalose-treated cells (Figure 4d). Trehalose also suppressed 6-OHDA-triggered mitochondrial depolarization in SH-SY5Y cells, as shown by the increase in the ratio of green to orange-red fluorescence (FL1/FL2) of the mitochondria-binding fluorochrome JC-1 (Figure 4e). The time kinetics of trehalose-mediated suppression of superoxide production and mitochondrial depolarization is shown in Supplementary Figure S2. Finally, TEM analysis revealed that the pretreatment with trehalose prevented the enlargement and swelling of mitochondria, as well as disorientation and partial or complete lysis of mitochondrial cristae in 6-OHDA-treated SH-SY5Y cells (Figure 4f). Therefore, trehalose protects SH-SY5Y cells from 6-OHDA by inhibiting oxidative stress, loss of mitochondrial membrane potential, and mitochondrial damage.

### 2.4. Trehalose-Mediated Protection Is Independent of Autophagy, p62, MAO-A and Antioxidant Enzyme Modulation

**Figure 5:**
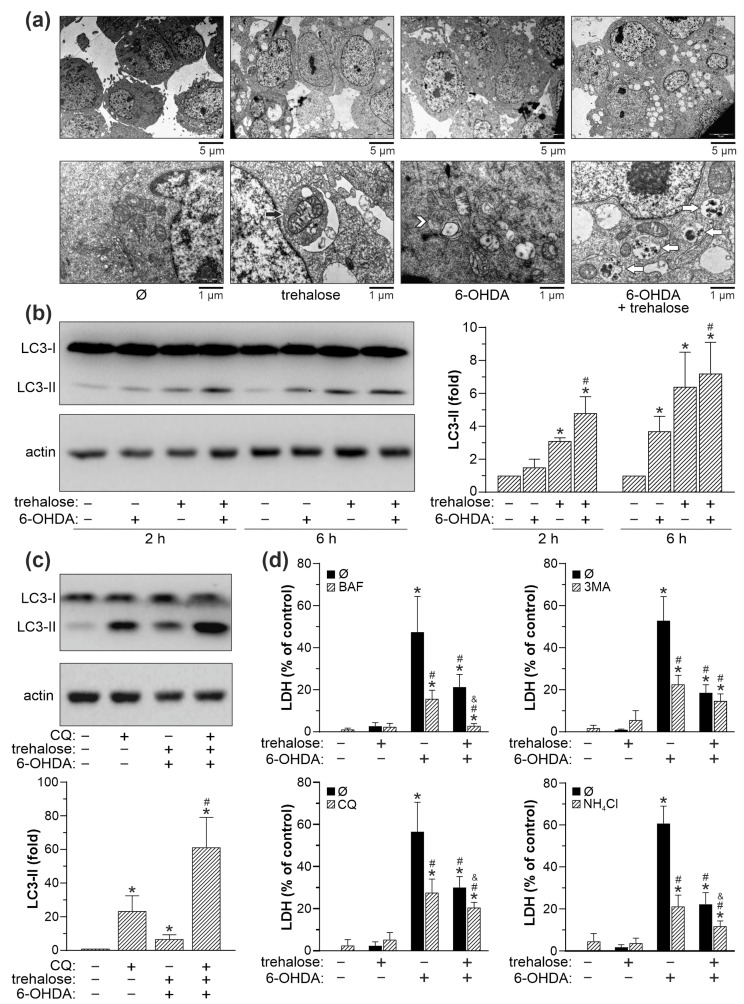
Trehalose-induced autophagy is not involved in protection from 6-OHDA. (**a**–**d**) SH-SY5Y cells were incubated with or without trehalose (100 mM) for 24 h and then treated or not with 6-OHDA (60 µM) for 8 h (**a**,**c**), the indicated times (**b**), or 24 h (**d**), in the presence or absence of chloroquine (CQ; 20 µM) (**c**,**d**), 3-methyladenine (3MA; 4 mM), bafilomycin A1 (BAF; 10 nM), or NH_4_Cl (10 mM) (**d**). The presence of autophagic vesicles was analyzed by TEM, showing double-membrane autophagosomes (white arrowhead) and single-membrane autolysosomes with cellular debris (white arrows), including mitochondria (black arrow) in 6-OHDA and/or trehalose-treated cells (**a**). The LC3 conversion was assessed by immunoblotting and quantified by densitometry (**b**,**c**), and the cytotoxicity was determined by LDH assay (**d**). The data in (**b**–**d**) are mean ± SD values from three independent experiments (* *p* < 0.05 vs. no treatment; # *p* < 0.05 vs. 6-OHDA; ^&^ *p* < 0.05 vs. 6-OHDA + trehalose). The representative TEM images (**a**) or immunoblots (**b**,**c**) from two (**a**) or three (**b**,**c**) independent experiments are shown.

**Figure 6:**
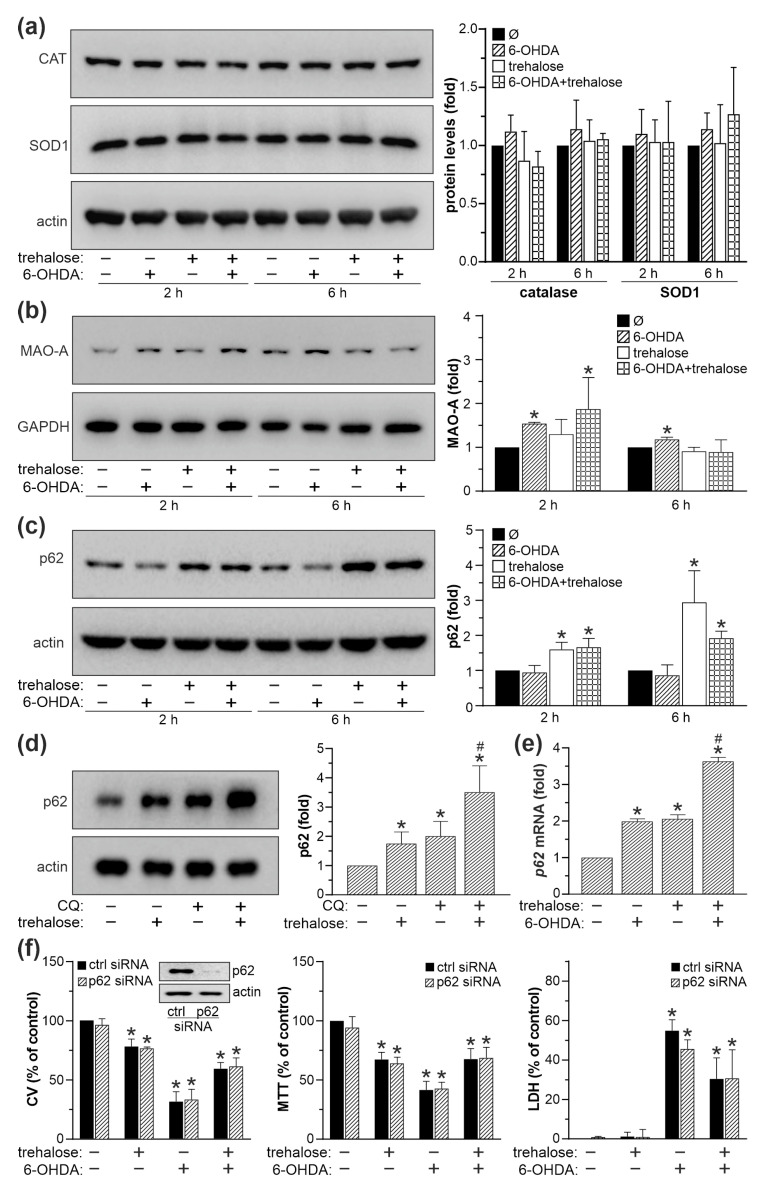
SOD1, catalase, MAO-A, and p62 are not involved in trehalose-mediated protection from 6-OHDA. (**a**–**d**) SH-SY5Y cells were incubated with or without trehalose (100 mM) for 24 h and then treated or not with 6-OHDA (60 µM) for the indicated times (**a**–**c**) or 6 h (**e**), or chloroquine (CQ; 20 µM) for 6 h (**d**). The protein levels of SOD1, catalase (**a**), MAO-A (**b**), and p62 (**c**,**d**) were assessed by immunoblotting and quantified by densitometry. The levels of *p62* mRNA were analyzed by RT-qPCR (**e**). (**f**) SH-SY5Y cells transfected with control or p62 siRNA were incubated with or without trehalose (100 mM) for 24 h and then treated or not with 6-OHDA (60 µM) for another 24 h. The inset shows a p62 decrease by siRNA. Cell viability was determined by crystal violet (CV) and MTT assays, and the cytotoxicity was assessed by LDH release. The data in (**a**–**f**) are mean ± SD values from three independent experiments (* *p* < 0.05 vs. no treatment; # *p* < 0.05 vs. 6-OHDA). The representative immunoblots are shown in (**a**–**d**,**f**).

We next investigated the role of autophagy, autophagic cargo receptor p62, MAO-A, and antioxidant enzymes superoxide dismutase 1 (SOD1) and catalase in trehalose-mediated protection of SH-SY5Y cells from 6-OHDA toxicity. TEM analysis revealed that both 6-OHDA and trehalose alone, and particularly when combined, increased the presence of autophagic vacuoles with partially digested cytoplasmic content (Figure 5a). Accordingly, immunoblot analysis demonstrated that trehalose, both alone and in combination with 6-OHDA, increased the conversion of cytoplasmic LC3-I to autophagosome-associated LC3-II form (Figure 5b). In the autophagic flux assay, the observed LC3-II increase in 6-OHDA + trehalose-treated cells was further augmented in the presence of the lysosomal proteolysis inhibitor chloroquine (Figure 5c), confirming that enhanced LC3 conversion, rather than the blockade of its proteolytic degradation, was responsible for LC3-II accumulation. Autophagic flux in 6-OHDA + trehalose treatment, calculated as the difference between 6-OHDA + trehalose-mediated LC3-II increase in the presence and absence of chloroquine, was 2.5-fold higher compared to untreated cells. Furthermore, the LDH assay showed that the inhibitors of autophagy induction (3-methyladenine) or lysosome-dependent autophagic degradation (bafilomycin A1, chloroquine, and ammonium chloride) reduced the cytotoxicity of 6-OHDA both in the absence and presence of trehalose (Figure 5d). Similar results were obtained using crystal violet and MTT assays (Supplementary Figure S3). These results indicate that autophagy is not involved in, and even counteracts the protective effect of trehalose in 6-OHDA-exposed SH-SY5Y cells. The preincubation of 6-OHDA-treated SH-SY5Y cells with trehalose failed to affect the protein levels of antioxidant enzymes SOD1 and catalase (Figure 6a), or MAO-A (Figure 6b), which converts 6-OHDA to various oxidative stress-inducing neurotoxic metabolites. On the other hand, trehalose alone and combined with 6-OHDA increased the protein levels of p62 (Figure 6c), an autophagic cargo receptor involved in cellular antioxidant defense [24]. The observed increase in p62 was preserved in the presence of the lysosomal proteolysis inhibitor chloroquine, indicating that de novo synthesis of p62, rather than the blockade of its autolysosomal proteolysis, was enhanced in trehalose-treated cells (Figure 6d). Accordingly, trehalose alone or in combination with 6-OHDA markedly increased the expression of *p62* mRNA in SH-SY5Y cells (Figure 6e). However, genetic inactivation of *p62* by siRNA failed to abolish the protective effect of trehalose in 6-OHDA-treated cells, as shown by crystal violet, MTT, and LDH assays (Figure 6f). Collectively, these results argue against the involvement of autophagy, p62, SOD1, catalase, or MAO-A in the protective effect of trehalose against 6-OHDA toxicity in SH-SY5Y cells.

### 2.5. Cytoprotective Effect of Trehalose Is Mediated by JNK, p38 MAPK, and AMPK Inhibition

**Figure 7:**
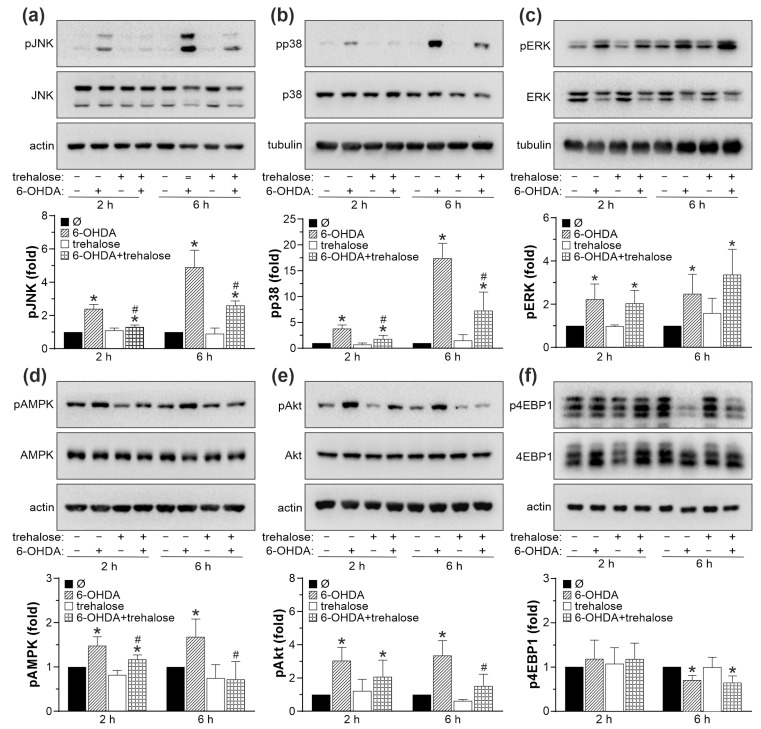
Trehalose inhibits 6-OHDA-induced activation of JNK, p38, and AMPK. (**a**–**f**) SH-SY5Y cells were incubated with or without trehalose (100 mM) and then treated or not with 6-OHDA (60 µM) for another 2 or 6 h. The levels of phosphorylated and total forms of JNK (**a**), p38 MAPK (**b**), ERK (**c**), AMPK (**d**), AKT (**e**), and 4EBP1 (**f**), together with loading controls (actin or tubulin), were assessed by immunoblotting, and the representative immunoblots are shown. Densitometry data are mean ± SD values from three independent experiments (* *p* < 0.05 vs. no treatment; # *p* < 0.05 vs. 6-OHDA).

To explore the possible involvement of cell death/survival-regulating protein kinases JNK, p38 MAPK, ERK, AMPK, AKT, and mTOR in the neuroprotective effects of trehalose, we first assessed if trehalose modulates their activity in 6-OHDA-treated SH-SY5Y cells. Immunoblot analysis demonstrated that 6-OHDA increased the levels of phosphorylated, active forms of JNK, p38 MAPK, ERK, and AMPK (Figure 7a–d). The phosphorylation of mTORC2 target AKT was also increased, while the phosphorylation of mTORC1 target 4EBP1 was decreased upon 6-OHDA treatment (Figure 7e,f). Trehalose significantly reduced the 6-OHDA-triggered increase in the levels of phosphorylated forms of JNK, p38 MAPK, and AMPK, both after 2 and 6 h of treatment (Figure 7a,b,d). Trehalose also reduced the phosphorylation of the mTORC2 substrate AKT in cells treated with 6-OHDA for 6 h (Figure 7e). The phosphorylation of ERK and mTORC1 substrate 4EBP1 in 6-OHDA-exposed cells was not affected by trehalose (Figure 7c,f).

**Figure 8:**
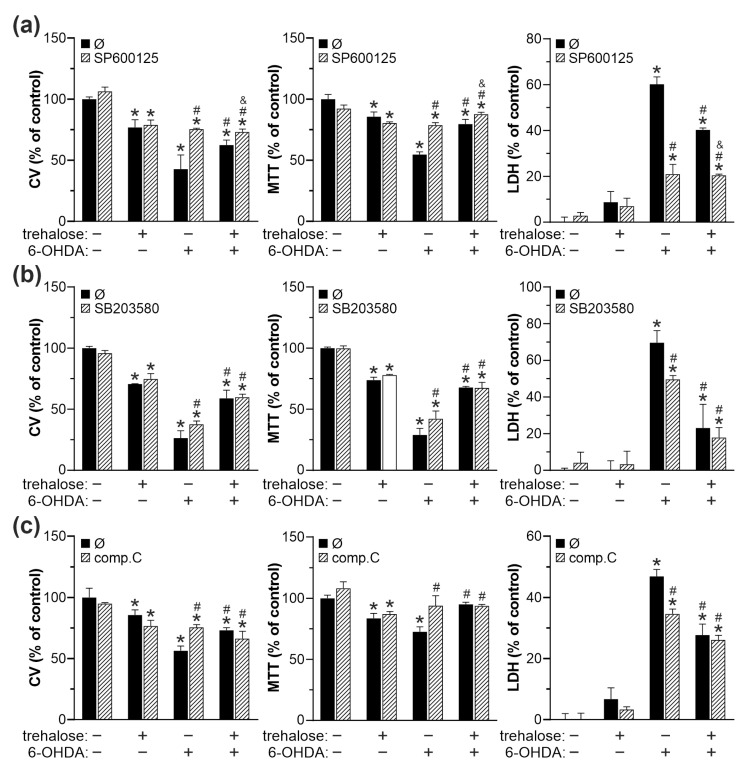
Inhibition of JNK, p38 MAPK, and AMPK is involved in trehalose-mediated protection from 6-OHDA. (**a**–**c**) SH-SY5Y cells were incubated with or without trehalose (100 mM) for 24 h and then treated or not with 6-OHDA (60 µM) for another 24 h, in the presence or absence of JNK inhibitor SP600125 (10 µM) (**a**), p38 MAPK inhibitor SB203580 (10 µM) (**b**), or AMPK inhibitor compound C (2 µM) (**c**). Cell viability was determined by crystal violet (CV) and MTT assays, and the cytotoxicity was evaluated by LDH release. The data are mean ± SD values of triplicate measurements from a representative of three experiments (* *p* < 0.05 vs. no treatment; # *p* < 0.05 vs. 6-OHDA; ^&^ *p* < 0.05 vs. 6-OHDA + trehalose).

We next used crystal violet, MTT, and LDH assays to test how pharmacological inhibition of JNK, p38 MAPK, and AMPK affects the protective action of trehalose. The ERK, mTORC1, and cytoprotective mTORC2/AKT were excluded from the analysis as they were unaltered (ERK, mTORC1/4EBP1) or reduced (mTORC2/AKT) by trehalose. SP600125, SB203580, and compound C, which inhibit JNK, p38 MAPK, and AMPK, respectively, each mimicked the cytoprotective action of trehalose in 6-OHDA-treated cells (Figure 8a–c). The protective effect of JNK, p38 MAPK, and AMPK inhibitors was less than additive with that of trehalose (Figure 8a–c). Similar results were obtained when JNK, p38 MAPK, or AMPK were downregulated by RNA interference (Supplementary Figures S4 and S5). Taken together, these results indicate that trehalose-mediated protection from 6-OHDA is ERK/mTORC1/2-independent and mediated by JNK, p38 MAPK, and AMPK suppression.

### 2.6. Trehalose Increases Oxidative Stress, Mitochondrial Damage, and Apoptotic Death in SH-SY5Y Cells Exposed to MPP^+^

**Figure 9:**
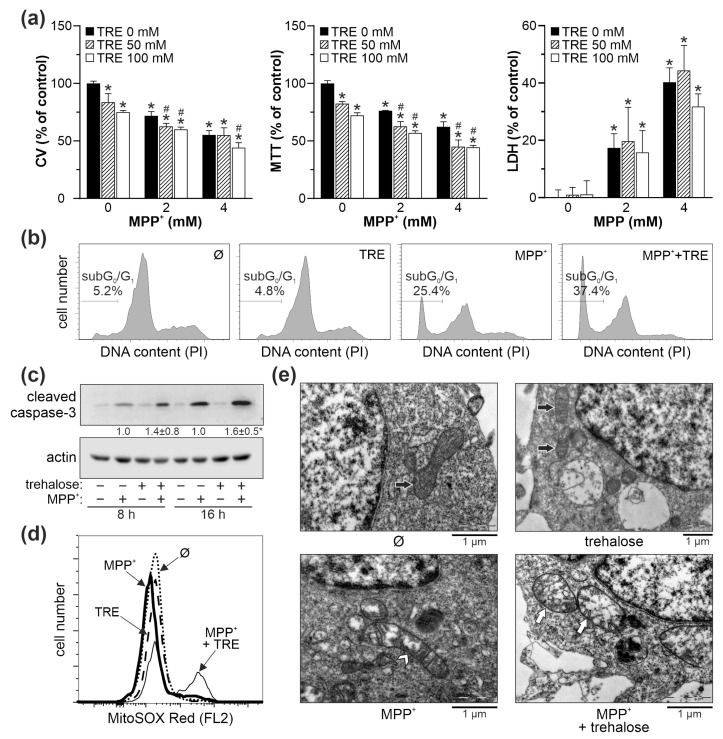
Trehalose increases MPP^+^-induced oxidative stress, mitochondrial damage, and apoptosis in SH-SY5Y cells. (**a**–**e**) SH-SY5Y cells were incubated with or without trehalose (TRE; 100 mM) for 24 h and then treated or not with the indicated doses (**a**) or 4 mM MPP^+^ (**b**–**e**) for 24 h (**a**,**b**), 8 h (**d**,**e**), or the indicated times (**c**). Cell viability was determined by crystal violet (CV) and MTT assays, and the cytotoxicity was assessed by LDH release (mean ± SD, n = 3; * *p* < 0.05 vs. no treatment; # *p* < 0.05 vs. MPP^+^) (**a**). DNA fragmentation was measured by flow cytometric analysis of PI-stained cells (**b**), and caspase-3 activation was analyzed by immunoblotting, with densitometry values below the relevant bands (mean ± SD, n = 3; * *p* < 0.05 vs. MPP^+^) (**c**). Intracellular ROS production was quantified by flow cytometric analysis of MitoSOX-stained cells (**d**). Mitochondrial morphology was visualized by TEM, showing normal mitochondria (black arrows), elongated mitochondria with partial cristolysis (white arrowhead), and swollen mitochondria with fragmented cristae (white arrows) (**e**). The representative flow cytometry histograms (**b**,**d**), immunoblots (**c**), and TEM images (**e**) from two (**b**,**d**,**e**) or three (**c**) independent experiments are shown.

Finally, to determine if trehalose protects against another parkinsonian toxin, MPP^+^, we incubated SH-SY5Y cells with 100 mM trehalose for 24 h and then exposed them to 2 mM or 4 mM of MPP^+^ for another 24 h. In contrast to the 6-OHDA treatment, trehalose further reduced the viability of MPP^+^-treated cells, as shown by the crystal violet and MTT assays, while no significant effects were observed on MPP^+^-induced LDH release (Figure 9a). Trehalose also increased apoptotic DNA fragmentation in MPP^+^-treated cells and activation of apoptosis-executing caspase-3, as demonstrated by flow cytometry and immunoblot, respectively (Figure 9b,c). Flow cytometric analysis of MitoSOX-stained cells showed that trehalose additionally increased superoxide production in MPP^+^-exposed cells (Figure 9d). TEM analysis of cells treated with MPP^+^ revealed elongated mitochondria with transverse cristae and small round mitochondria with disoriented cristae, both characterized by partial cristolysis and a moderately electron-lucent matrix (Figure 9e). The cells treated with both trehalose and MPP^+^ displayed a more pronounced mitochondrial damage characterized by large, swollen mitochondria with fragmented/disorganized cristae and a very electron-lucent matrix (Figure 9e).

**Figure 10:**
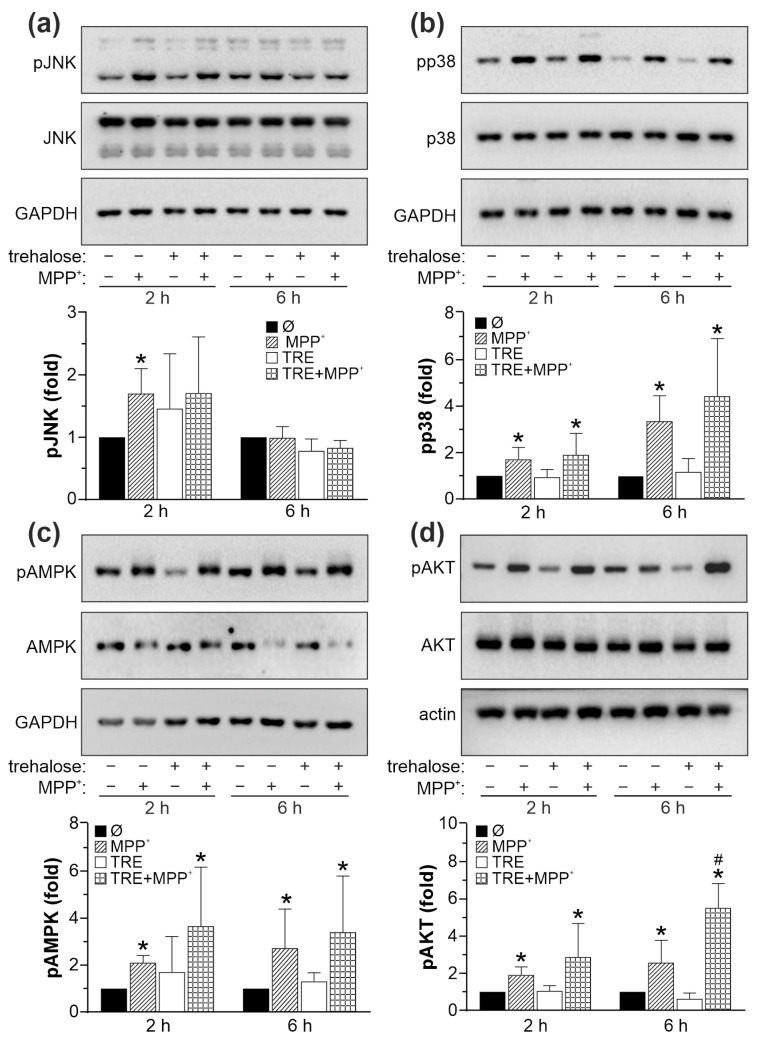
The effects of trehalose on MAPK/AMPK and AKT signaling in MPP+-treated SH-SY5Y cells. (**a**–**d**) SH-SY5Y cells were incubated with or without trehalose (TRE; 100 mM) and then treated or not with MPP^+^ (4 mM) for another 2 or 6 h. The levels of phosphorylated and total forms of JNK (**a**), p38 MAPK (**b**), AMPK (**c**), and AKT (**d**), together with loading controls (GAPDH or actin), were assessed by immunoblotting, and the representative immunoblots are shown. Densitometry data are mean ± SD values from three independent experiments (* *p* < 0.05 vs. no treatment; # *p* < 0.05 vs. MPP^+^).

Similar to 6-OHDA, MPP^+^ increased the phosphorylation of JNK, p38 MAPK, AMPK, and AKT in SH-SY5Y cells (Figure 10). However, trehalose failed to modulate the activation of JNK, p38 MAPK, and AMPK in MPP^+^-treated cells (Figure 10a–c), while increasing the activation of the prosurvival mTORC2 target AKT at the later time point (Figure 10d). Thus, in contrast to the protective action in 6-OHDA-exposed cells, trehalose potentiated the neurotoxicity of MPP^+^ by stimulating oxidative stress, mitochondrial damage, and apoptosis independently of JNK, p38 MAPK, AMPK, or AKT signaling.

## 3. Discussion

We here demonstrate that trehalose reduces the in vitro toxicity of the parkinsonian mimetic 6-OHDA through autophagy/p62-independent suppression of oxidative stress, mitochondrial depolarization/damage, and caspase-dependent apoptosis. The observed protection was exerted through modulation of JNK, p38 MAPK, and AMPK but not ERK or mTORC1/2 signaling pathways. The effect was selective for 6-OHDA, as trehalose increased the oxidative stress and mitochondrial dysfunction induced by another neurotoxin, MPP^+^. These findings provide additional mechanistic insight into the trehalose’s previously reported protective/detrimental actions in the 6-OHDA and MPP^+^ neurotoxicity [19,20,21,22,23].

Trehalose exerts neuroprotective effects through both autophagy-dependent and autophagy-independent mechanisms [12]. In our experiments, trehalose increased the autophagic flux in SH-SY5Y cells, while pharmacological inhibitors of autophagy reduced 6-OHDA cytotoxicity and enhanced the protective effect of trehalose. These results confirm the detrimental role of excessive autophagy/mitophagy in 6-OHDA neurotoxicity [25,26,27,28,29,30,31], implying that autophagy is not involved in, and might even oppose trehalose-mediated cytoprotection. Of note, autophagy counteracted 6-OHDA neurotoxicity in some studies [32,33,34,35,36], possibly due to differences in target cell type, autophagy modulators, exposure times, and/or neurotoxin concentrations. While these discrepancies remain to be resolved, our findings suggest that trehalose-mediated neuroprotection from 6-OHDA could benefit from an additional blockade of autophagy.

ROS generated by autoxidation or MAO-A-catalyzed degradation of 6-OHDA impair mitochondrial function and induce apoptosis through oxidative damage of proteins, lipids, and nucleic acids [2]. In our study, trehalose reduced 6-OHDA-triggered ROS production and mitochondrial injury, thus attenuating the vicious circle of oxidative stress and mitochondrial dysfunction. Trehalose was previously found to reduce oxidative stress in epididymal/renal tubule epithelial cells by increasing the expression/activation of antioxidant enzymes [37,38]. Moreover, trehalose-mediated protection from 6-OHDA neurotoxicity in vivo was associated with the increase in p62 levels, NRF2 nuclear translocation, and upregulation of glutathione peroxidase, glutathione reductase, and catalase [19], but the connection between these events was not directly examined. Despite stimulating autophagic flux, trehalose in our experiments increased the intracellular levels of p62 through a transcription-dependent de novo synthesis. However, trehalose did not alter the levels of catalase/SOD1, and p62 knockdown failed to affect its protective action, arguing against the role of the p62/NRF2 antioxidant axis in our experimental system. As the ROS production in the cell-free solution of 6-OHDA was unaffected by trehalose, it is unlikely that direct ROS scavenging significantly contributed to the observed cytoprotection. Also, the inability of trehalose to modulate the expression of MAO-A indicates that its antioxidative/antiapoptotic activity was not exerted via interference with MAO-mediated conversion of 6-OHDA to cytotoxic ROS.

The intracellular energy sensor AMPK and MAPK family members JNK/p38 contribute to the in vitro cytotoxicity of 6-OHDA [25,39,40,41,42,43,44,45], as confirmed here. Moreover, our data indicate that the inhibition of JNK, p38 MAPK, and AMPK was involved in the protective action of trehalose. This assumption is reinforced by a less than additive protection upon combining trehalose with the kinase inhibitors, indicating a common protective mechanism. Accordingly, the inhibition of JNK, p38, or AMPK was associated with trehalose-mediated protection from H_2_O_2_-induced death of SH-SY5Y cells [15], LPS-triggered oxidative damage in leukocytes [16], insulin resistance-induced apoptosis in cardiomyocytes [17], desiccation stress in corneal cells [46], and age-related degeneration of retinal cells [47]. The ERK and mTORC1/2 pathways play a dual role in 6-OHDA cytotoxicity. While rapid activation of ERK is neuroprotective [48], its sustained activity contributes to the demise of neuronal cells [31,49]. Similarly, the mTOR pathway incorporates both neurotoxic and neuroprotective signaling branches mediated by mTORC1 and mTORC2/AKT, respectively [31,50,51,52]. However, the inability of trehalose to inhibit neurotoxic ERK/mTORC1 and/or activate neuroprotective mTORC2/AKT pathways argues against their involvement in trehalose-mediated protection from 6-OHDA.

There is a question about the molecular mechanisms underlying trehalose-mediated cytoprotection, decrease of oxidative stress, and suppression of MAPK and AMPK signaling pathways in 6-OHDA-exposed cells. The key point in this context is probably the antioxidant activity of trehalose, as oxidative stress can damage neurons both directly, through oxidative damage of macromolecules [53], and indirectly, through activation of cell death signaling pathways such as MAPK and AMPK [54]. Our preliminary data indicate that protein synthesis was required for the cytoprotective action of trehalose, which was reduced if the translation was blocked with cycloheximide during trehalose pretreatment. As we excluded the role of p62, SOD1, catalase, or MAO-A modulation in the trehalose pro-survival effect, our research is currently focused on the molecule(s) involved in the observed protein synthesis-sensitive cytoprotection. Also, since JNK activation and translocation to mitochondria triggers mitochondrial dysfunction and oxidative stress [55], it would be interesting to examine the possible effects of trehalose on the positive feedback interaction between ROS and JNK.

Finally, we show in the same experimental setting that, in contrast to its effects in 6-OHDA-treated SH-SY5Y cells, trehalose potentiated oxidative stress, mitochondrial damage, and apoptotic cell death induced by another neurotoxin, MPP^+^. This is consistent with a trehalose-mediated increase in MPP^+^ toxicity towards SK-N-SH neuroblastoma cells [22,23], from which the SH-SY5Y cell line was subcloned. The opposing action of trehalose on 6-OHDA and MPP^+^ cytotoxicity could be due to different mechanisms of mitochondrial dysfunction and oxidative stress induced by the two parkinsonian toxins. MPP^+^ accumulates in mitochondria where it directly targets complex I of the electron transport chain and increases ROS generation as a result [56], with the subsequent impairment of energy metabolism, rather than oxidative stress, being a primary cause of toxicity [57,58]. On the other hand, although 6-OHDA inhibits complexes I and IV in isolated mitochondria [59], its cytotoxic ability mainly depends on ROS generated via extracellular and intracellular autoxidation [60,61,62,63]. This suggests an intriguing possibility that trehalose might prevent mitochondrial dysfunction and cell death by targeting 6-OHDA-mediated ROS generation, while not being able to counteract direct inhibition of mitochondrial complex I and subsequent ROS increase by MPP^+^. The inability of trehalose to reduce MPP^+^-triggered oxidative stress is consistent with no decrease in ROS-dependent intracellular signaling pathways, such as JNK, p38 MAPK, AMPK, and AKT. Although unable to explain the increase of MPP^+^ toxicity by trehalose, this finding indirectly argues for the role of ROS decrease in trehalose-mediated signaling perturbations in 6-OHDA treatment. We currently investigate the mechanisms of trehalose-induced potentiation of MPP^+^ cytotoxicity, including the possible role of autophagy.

The limitations of the present study include the use of undifferentiated SH-SY5Y neuroblastoma cells and the in vitro treatment with neurotoxins. While there are contradicting findings on the sensitivity of undifferentiated vs. differentiated SH-SY5Y cells to parkinsonian toxins [64,65,66], the latter better reflects the responses of terminally differentiated dopaminergic neurons [67]. Also, the in vivo neurotoxin-based and genetic models, especially in combination, more faithfully recapitulate neuronal PD pathology. Lastly, although trehalose crosses the blood-brain barrier and can be administered nasally to bypass it in animal models [68,69], its safety and effectiveness in humans remain to be evaluated. Nevertheless, we contend that the present report lays a sound foundation for addressing these issues in further studies.

In conclusion, the protective effect of trehalose against 6-OHDA-induced oxidative stress, mitochondrial damage, and apoptosis relies on autophagy/p62-independent antioxidant and MAPK/AMPK-inhibiting actions. While the contrasting effects of trehalose in 6-OHDA vs. MPP^+^ treatment reflect their different neurotoxic mechanisms, it is important to note that neither of the two parkinsonian mimetics replicates the entire spectrum of pathological and clinical features of PD [70]. Even so, the potentiation of MPP^+^-induced apoptosis indicates that further exploration of trehalose as a neuroprotective agent, including the ongoing PD clinical trials (NCT05355064), should proceed with due caution. Future studies will hopefully provide further mechanistic insight into trehalose’s beneficial and detrimental effects on neuronal survival in different in vitro and in vivo experimental settings.

## 4. Materials and Methods

### 4.1. Cell Culture and Treatments

The human neuroblastoma cell line SH-SY5Y was obtained from the American Type Culture Collection (ATCC CRL-2266) and grown in Modified Eagle Medium (Thermo Fisher Scientific, Waltham, MA, USA) and Ham’s Nutrient Mixture F12 (HAM-12-A; Capricorn Scientific, Ebsdorfergrund, Germany) at a ratio of 1:1, supplemented with 10% fetal calf serum (FCS), 2 mM L-glutamine, 1% nonessential amino acids, and 1% penicillin/streptomycin (all from Merck KgaA, Darmstadt, Germany). Cells were incubated in a 5% CO_2_ humidified atmosphere at 37 °C and prepared for experiments using the conventional trypsinization procedure with trypsin/ethylenediaminetetraacetic acid (EDTA) (Merck KgaA, Darmstadt, Germany). Cells were incubated in 96-well flat-bottom plates (3 × 10^4^ cells/well) for viability assays, 24-well plates (1 × 10^5^ cells/well) for flow cytometric analysis, 60 mm Petri dishes (2 × 10^6^ cells) for electron microscopy and immunoblotting, and 175 cm^2^ flasks (10 × 10^6^ cells) for determination of reduced glutathione (all cell culture plastic from Sarstedt AG & Co., Nümbrecht, Germany). Before experiments, cells were rested in a cell culture medium for 24 h and then incubated for 0.5–24 h with trehalose, followed by the addition of 6-OHDA or MPP^+^ (all from Merck KgaA, Darmstadt, Germany). In some experiments, autophagy inhibitors 3-methyladenine, bafilomycin A1, chloroquine, and ammonium chloride, JNK inhibitor SP600125 [71], p38 MAPK inhibitor SB203580 [72], or AMPK inhibitor compound C [73] (all from Merck KgaA, Darmstadt, Germany) were added 30 min before 6-OHDA. Trehalose, 3-methyladenine, and ammonium chloride were dissolved directly in the cell culture medium. The stock solutions of bafilomycin A1, chloroquine, SP600125, SB203580, and compound C were made in dimethyl sulfoxide (DMSO) (Merck KgaA, Darmstadt, Germany), and the final concentrations of DMSO in cell culture were 0.01%, 0.05%, 0.01%, 0.02%, and 0.02%, respectively. Control cell cultures without pharmacological inhibitors contained the corresponding amount of DMSO. The concentrations of autophagy and signaling inhibitors used in experiments were chosen based on the previous reports [25,74,75].

### 4.2. Cell Viability Assays

Cell viability was determined by measuring the number of adherent cells and mitochondrial dehydrogenase activity using the crystal violet and MTT reduction assays, respectively, as previously described [76]. Crystal violet and MTT were obtained from Merck KgaA, Darmstadt, Germany. The light absorbance of crystal violet (dissolved in 33% acetic acid) and MTT formazan crystals (dissolved in DMSO), corresponding to the number of viable cells, was measured in a colorimetric microplate reader (Tecan, Dorset, UK) at 570 nm. The results of both assays were expressed as the percentage of cell viability relative to the control value of untreated cells, which was arbitrarily set to 100%.

### 4.3. LDH Release Cytotoxicity Assay

Cell membrane damage, as a cell death marker, was assessed by measuring the release of the intracellular enzyme LDH into the cell culture medium. Briefly, 50 μL of cell culture supernatant was mixed with 50 μL of solution containing 54 mM lactic acid, 0.28 mM phenazine methosulfate, 0.66 mM p-iodonitrotetrazolium chloride, and 1.3 mM β-nicotinamide-adenine dinucleotide (NAD) (all from Merck KgaA, Darmstadt, Germany). LDH activity is quantified by using the NADH produced during the conversion of lactate to pyruvate to reduce p-iodonitrotetrazolium chloride into a red formazan, whose absorbance at 492 nm was measured by a colorimetric microplate reader (Tecan, Dorset, UK). The percentage of dead cells was determined using the following formula: [(E − C)/(T − C)] × 100, where E is the experimental absorbance of treated cells, C is the control absorbance of untreated cells, and T is the absorbance corresponding to the maximal (100%) LDH release of Triton X-100-lysed cells. To avoid underestimating LDH release due to trehalose-mediated growth inhibition, we used cells incubated with trehalose alone as maximal LDH release controls for 6-OHDA + trehalose-treated cells [77].

### 4.4. Cell Cycle and Apoptosis Analysis

Cell cycle and DNA fragmentation/phosphatidylserine externalization in apoptotic cells were measured by flow cytometry. For the cell cycle and DNA fragmentation analysis, cells were centrifuged at 700× *g* for 10 min and washed twice in phosphate buffered saline (PBS) with 2% FCS and 2 mM EDTA (all from Merck KgaA, Darmstadt, Germany). Cells were then incubated with RNAse A (50 µg/mL) and DNA-intercalating dye propidium iodide (50 µg/mL) (both from Thermo Fisher Scientific, Waltham, MA, USA) dissolved in sodium citrate buffer (0.1% sodium citrate, 0.1% Triton X-100 in dH_2_O) (Merck KgaA, Darmstadt, Germany) at 4 °C for 30 min. The proportion of cells in various phases of the cell cycle, including hypodiploid apoptotic cells in sub-G_0_/G_1_, was determined by measuring the orange-red fluorescence (FL2 channel), using FL2-W vs. FL2-A dot plot to exclude cell aggregates. Phosphatidylserine externalization was analyzed following double staining with annexin V-fluorescein isothiocyanate (FITC; FL1) and propidium iodide (FL2), in which annexin V binds to early apoptotic cells with exposed phosphatidylserine, while propidium iodide labels the late apoptotic/necrotic cells with membrane damage. Staining was performed according to the instructions by the manufacturer (BD Biosciences, San Diego, CA, USA). All flow cytometry measurements were performed on the FACS Aria III flow cytometer (BD Biosciences, San Diego, CA, USA), using FACSDIVA 6.0 software for acquisition and FlowJo 10.7 software for analysis (BD Biosciences, San Diego, CA, USA).

### 4.5. Transmission Electron Microscopy (TEM)

For the TEM analysis of ultrastructural morphology, cells were trypsinized in 3% glutaraldehyde, postfixed in 1% osmium tetroxide, dehydrated in graded alcohols, and then embedded in Epon 812 (all from Merck KgaA, Darmstadt, Germany). Ultrathin sections were stained in uranyl acetate and lead citrate (both from Merck KgaA, Darmstadt, Germany) and examined with a Morgagni 268D electron microscope (FEI, Hillsboro, OR, USA). Images were acquired using a MegaView III CCD camera and iTEM 5.0 (build 1243) software (Olympus Soft Imaging Solutions, Münster, Germany).

### 4.6. Determination of Reduced Glutathione

Cellular redox status was determined by spectrophotometric measurement of reduced glutathione (GSH). Briefly, 5 × 10^6^ cells were resuspended in 30 µL of 10% (*w*/*v*) sulfosalicylic acid (Merck KgaA, Darmstadt, Germany), mixed by vortexing, and centrifuged at 10,000× *g* at +4 °C for 10 min to remove protein precipitate. The supernatant (50 µL) was then mixed with 50 µL of reaction reagent prepared by dissolving 10 mM 5,5-dithiobis(2-nitrobenzoic acid) in TRIS-HCl pH 8.9 (both from Merck KgaA, Darmstadt, Germany). During incubation at room temperature in the dark for 20 min, GSH reduced 5,5-dithiobis(2-nitrobenzoic acid) to 5′-thio-2-nitrobenzoic acid, whose light absorbance at 405 nm was measured using a colorimetric microplate reader (Tecan, Dorset, UK). The GSH concentration was calculated using the GSH (Merck KgaA, Darmstadt, Germany) standard curve.

### 4.7. Measurement of Reactive Oxygen Species (ROS) and Mitochondrial Depolarization

The total intracellular ROS production and mitochondrial superoxide generation were determined by measuring the fluorescence intensity emitted by dihydrorhodamine (DHR) and MitoSOX Red (both from Thermo Fisher Scientific, Waltham, MA, USA), respectively. Various ROS oxidize DHR to its green-fluorescent derivative rhodamine 123, while MitoSOX Red accumulates in mitochondria, exhibiting red fluorescence upon oxidation by mitochondrial superoxide. Briefly, DHR (2 μM) was added to the cell cultures at the beginning of treatment, while MitoSOX Red (5 μM) was added during the last 10 min of treatment. At the end of incubation, cells were detached by trypsinization and washed in PBS. The median intensity of DHR green fluorescence (FL1) or MitoSOX orange-red fluorescence (FL2), representing total ROS production or mitochondrial superoxide generation, respectively, was quantified by flow cytometry. The results were presented as the fold change relative to the median FL1 (DHR) or FL2 (MitoSOX) value of the untreated cells, which was arbitrarily set to 1. Alternatively, DHR fluorescence (excitation 488 nm, detection 535 nm) was used to determine ROS generation by 6-OHDA in cell-free conditions, using Synergy H1 Hybrid Multi-Mode Reader (BioTek Instruments, Winooski, VT, USA). Mitochondrial membrane potential was measured using JC-1 (Trevigen Inc., Gaithersburg, MD, USA), a lipophilic cation that aggregates upon mitochondrial membrane polarization, forming an orange-red fluorescent compound. When the membrane potential is disrupted, the dye cannot access the transmembrane space and remains or reverts to its green monomeric form. Cells were detached by trypsinization and stained with JC-1 (5 μg/mL in a JC-1 staining buffer) for 20 min at 37 °C. The green monomers and red aggregates were detected by flow cytometry. The results are presented as the fold change in green/orange-red fluorescence ratio (median FL1/FL2, arbitrarily set to 1 in control samples), with the increase in FL1/FL2 reflecting mitochondrial depolarization. All flow cytometry measurements (DHR, MitoSOX, JC-1) were performed on the FACS Aria III flow cytometer (BD Biosciences, San Diego, CA, USA), using FACSDIVA 6.0 software for acquisition and FlowJo 10.7 software for analysis (BD Biosciences, San Diego, CA, USA).

### 4.8. Quantitative Reverse Transcription Polymerase Chain Reaction (RT-qPCR)

The RT-qPCR was used to determine *SQSTM1* (*p62*) mRNA levels. After extraction with TRIzol/chloroform (Invitrogen, Carlsbad, CA, USA), isopropanol precipitation, and washing in ethanol, total cellular RNA (1 μg) was reverse transcribed with MuLV reverse transcriptase and oligo(dT) primers (both from Thermo Fisher Scientific, Waltham, MA, USA), following the manufacturer’s protocol. The PCR amplification was performed in a Realplex^2^ Mastercycler (Eppendorf, Hamburg, Germany) using 96-well reaction plates, Maxima Hot Start PCR Master Mix, and TaqMan primers/probes (all from Thermo Fisher Scientific, Waltham, MA, USA)for human *p62*/*SQSTM1* (Hs00177654_m1), while TATA box binding protein (*TBP*; Hs99999910_m1) and hypoxanthine phosphoribosyltransferase 1 (*HPRT1*; Hs02800695_m1) were used as housekeeping genes. The thermal cycle conditions were 95 °C for 4 min, followed by 40 cycles of 15 s at 95 °C, and 1 min at 60 °C. All reactions were performed in triplicates. The average geomean cycle of threshold (Ct) values of *TAB*/*HPRT1* were subtracted from the average *p62* Ct values to obtain ΔCt, and the ΔΔCt values were obtained by subtracting the ΔCt values of the untreated control sample from the ΔCt values of the treated samples. The relative gene expression was determined as 2^−ΔΔCt^, and the results were presented as the fold change relative to gene expression in the untreated control sample.

### 4.9. Immunoblotting

Immunoblotting was used to analyze the expression/phosphorylation of specific proteins in cell lysates. After washing with PBS, cells were lysed in lysis buffer (30 mM Tris-HCl pH 8.0, 150 mM NaCl, 1% Nonidet P-40, and protease/phosphatase inhibitor mixture) (all from Merck KgaA, Darmstadt, Germany) on ice for 30 min. The cell lysates were centrifuged at 14,000× *g* for 15 min at 4 °C, and the supernatants were collected. Equal amounts of denatured proteins from each sample were separated by SDS-PAGE and transferred to nitrocellulose membranes (Bio-Rad, Hercules, CA, USA). After blocking with 5% nonfat dried milk or bovine serum albumin (for anti-phosphoprotein antibodies), the membranes were incubated overnight at 4 °C with primary rabbit anti-human antibodies against PARP1, cleaved caspase-3, LC3B, JNK1/2, phospho-JNK1/2 (Thr183/Tyr185), ERK1/2, phospho-ERK1/2 (Thr202/Tyr204), p38 MAPK, phospho-p38 MAPK (Thr180/Tyr182), AMPKα1/2, phospho-AMPKα1/2 (Thr172), AKT, phospho-AKT (Ser473), 4EBP1, phospho-4EBP1 (Thr37/46), catalase, (all from Cell Signaling Technology, Beverly, MA, USA), SOD1 (Santa Cruz Biotechnology, Santa Cruz, CA, USA), MAO-A (Abcam, Cambridge, UK), sequestosome 1/p62, β-actin (both from Novus Biologicals, Littleton, CO, USA), glyceraldehyde 3 phosphate dehydrogenase (GAPDH; Thermo Fisher Scientific, Waltham, MA, USA), or primary mouse anti-human antibody against β-tubulin (Proteintech, Rosemont, IL, USA). A peroxidase-conjugated goat anti-rabbit IgG (Jackson IP Laboratories, West Grove, PA, USA) and goat anti-mouse IgG (Southern Biotech, Birmingham, AL, USA) were used as secondary antibodies. The antibody dilutions and incubation times were as recommended by the manufacturers. The specific protein bands were visualized using a chemiluminescence solution (0.24 mM p-coumaric acid, 1.5 mM luminol, 0.009% H_2_O_2_) (all from Merck KgaA, Darmstadt, Germany) on a ChemiDoc Imaging System (Bio-Rad Laboratories, Hercules, CA, USA), and the signal intensity was quantified by densitometry using the Image Lab 5.0 software (Bio-Rad Laboratories, Hercules, CA, USA). The protein levels of caspase-3, PARP1, catalase, SOD1, LC3-II, and p62 were expressed relative to the loading control, while the levels of phosphorylated proteins were expressed relative to the total amount of the corresponding protein. The results are presented as a fold change relative to the densitometry signal of the untreated control sample.

### 4.10. RNA Interference

Small interfering RNA (siRNA) targeting human p62, JNK, AMPK, p38 MAPK and corresponding control siRNA (all from Santa Cruz Biotechnology, Santa Cruz, CA, USA) were transfected into SH-SY5Y cells by electroporation in the 4D-Nucleofector X Unit, using the SF Cell Line 4D-Nucleofector X Kit and CA-137 program (Lonza, Basel, Switzerland), according to the manufacturer’s instructions. After transfection, the cells were rested for 16 h before treatment.

### 4.11. Statistical Analysis

The data are presented as mean ± SD values from at least three independent experiments or triplicates from a representative experiment, as indicated in the figure legends. While the data are shown as fold/percent change values to increase clarity, the statistical analysis was performed on the original data. As recommended for very small (n ≤ 5) sample sizes [78], the statistical significance of the differences between treatments was assessed using a two-tailed paired or unpaired t-test for the data obtained from independent experiments or multiple observations in the same experiment, respectively. A *p*-value of less than 0.05 was considered statistically significant. To reduce the risk of missing the true effects, no corrections for multiple comparisons were made [79].

## Figures and Tables

**Table 1:** The effects of trehalose on SH-SY5Y cell numbers and viability.

	Crystal Violet (%)		MTT (%)		LDH Release (%)	
TRE (mM)	24 h	48 h	24 h	48 h	24 h	48 h
0	100	100	100	100	0	0
50	96.3 ± 6.5	82.8 ± 4.1 *	85.4 ± 8.9 *	78.0 ± 4.4 *	4.6 ± 3.1	3.0 ± 4.0
100	87.8 ± 4.3 *	77.1 ± 3.8 *	74.6 ± 7.4 *	66.6 ± 5.4 *	4.2 ± 2.7	3.1 ± 4.3
200	67.1 ± 7.0 *	46.2 ± 4.4 *	59.6 ± 4.7 *	39.8 ± 5.1 *	17.2 ± 0.6 *	35.4 ± 0.3 *

* *p* < 0.05 vs. no trehalose (data are mean ± SD values from three independent experiments); TRE, trehalose.

## Data Availability

The original contributions presented in the study are included in the article/Supplementary Materials, further inquiries can be directed to the corresponding authors.

## Supplementary Materials

The following supporting information can be downloaded at: https://www.mdpi.com/article/10.3390/ijms251910659/s1.
