# Supplementary material for: Trehalose Attenuates In Vitro Neurotoxicity of 6-Hydroxydopamine by Reducing Oxidative Stress and Activation of MAPK/AMPK Signaling Pathways
Source: Int J Mol Sci. 2024 Oct 3;25(19):10659. doi: 10.3390/ijms251910659 (PMC11476739; doi:10.3390/ijms251910659)
Supplement: Supplementary file 1 [file ijms-25-10659-s001.zip › ijms-3209994-supplementary.pdf]

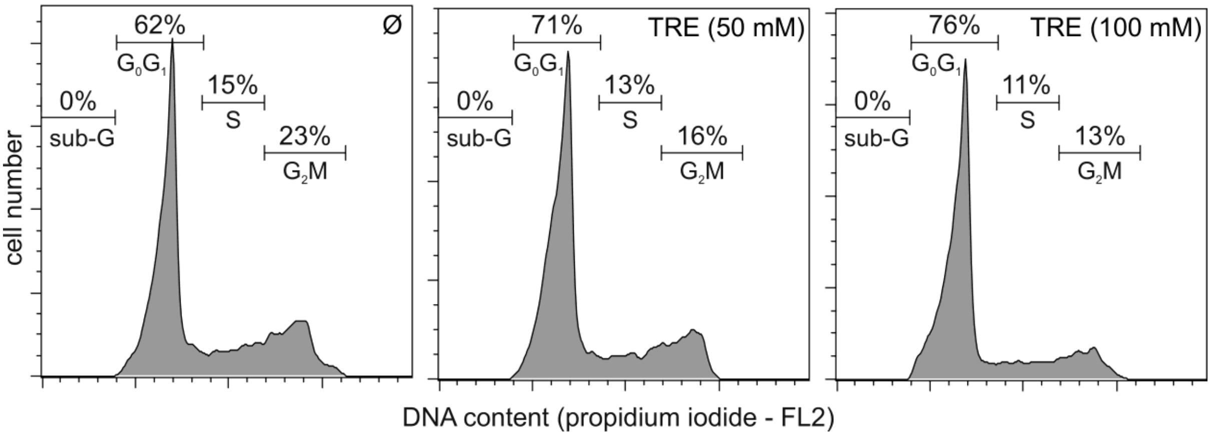

Figure S1. Trehalose induces cell cycle block in SH-SY5Y cells. SH-SY5Y cells were incubated with or without trehalose (50 or 100 mM) for 48 h, and the cell cycle phases in propidium iodide-stained cells were analyzed by flow cytometry. The results from a representative of three independent experiments are presented.

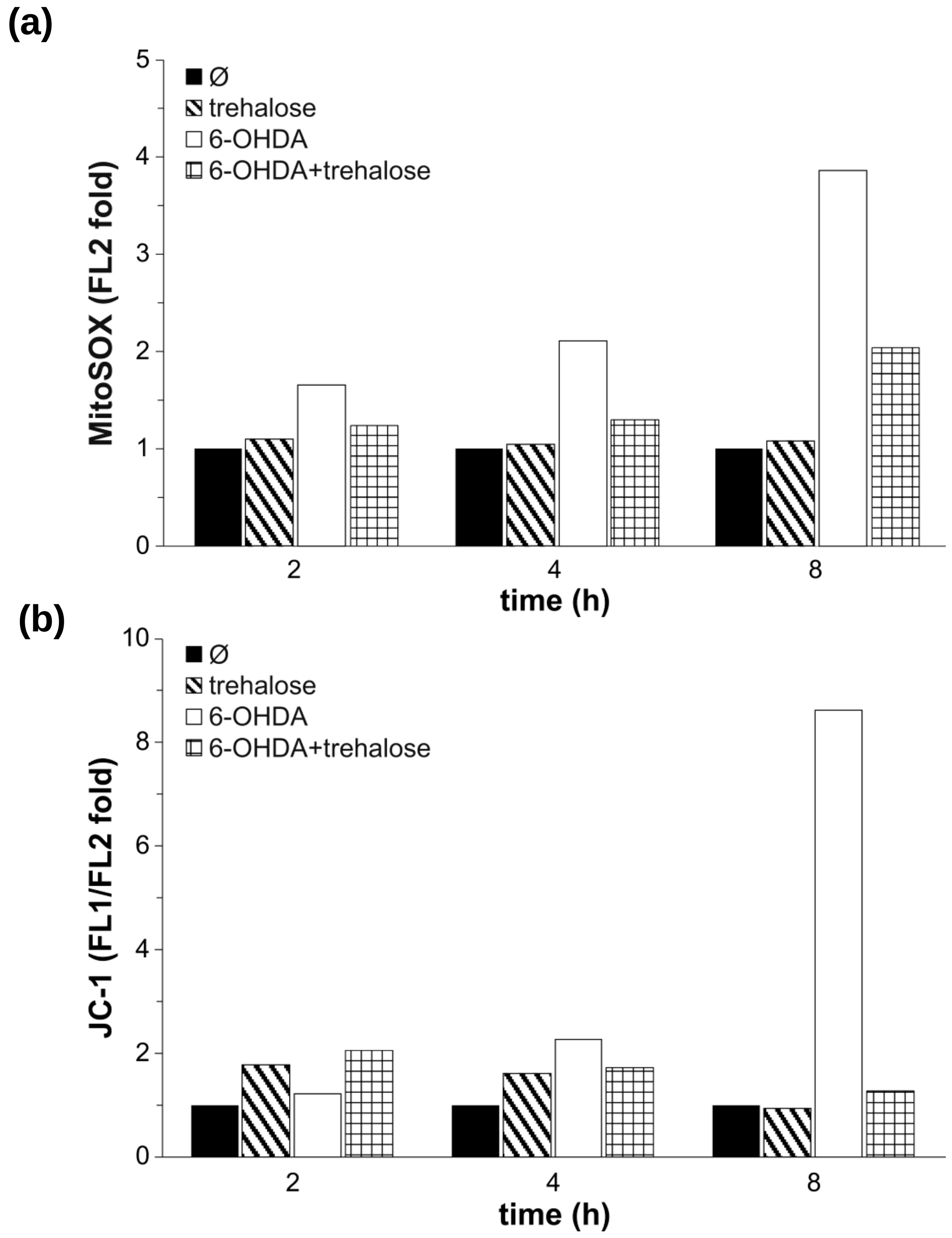

Figure S2. Trehalose prevents 6-OHDA-induced mitochondrial superoxide production and depolarization in SH-SY5Y cells. SH-SY5Y cells were incubated with or without trehalose (TRE; 100 mM) for 24 h and then exposed or not to 6-OHDA (60 µM) for the indicated times. Mitochondrial superoxide production (**a**), and mitochondrial depolarization (**b**) were determined by flow cytometric analysis of MitoSOX (FL2) and JC-1 (FL1/Fl2) median fluorescence, respectively. The data from a representative of two independent experiments are shown.

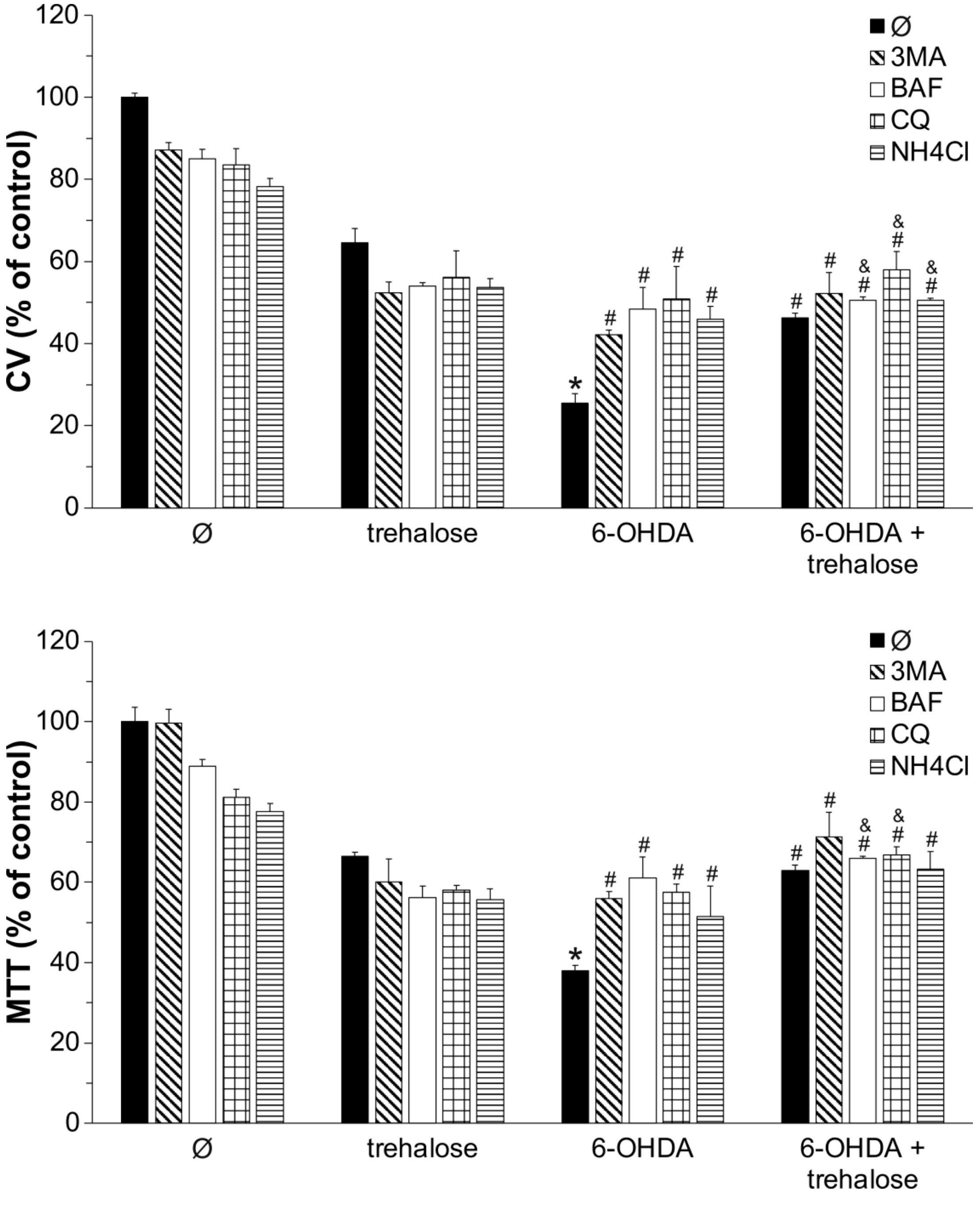

Figure S3. Autophagy is not involved in trehalose-mediated protection from 6-OHDA. SH-SY5Y cells were incubated with or without trehalose (100 mM) for 24 h and then treated or not with 6-OHDA (60 µM) for 24 h, in the presence or absence of 3-methyladenine (3MA; 4 mM), bafilomycin A1 (BAF; 10 nM), chloroquine (CQ; 20 µM), or NH4Cl (10 mM). Cell viability was determined by crystal violet (CV) and MTT assays, and the cytotoxicity was evaluated by LDH release. The data are presented as mean ± SD values of triplicates from a representative of three independent experiments (*p < 0.05 vs. no treatment; #p < 0.05 vs. 6-OHDA; $^{\&}$p < 0.05 vs. 6-OHDA + trehalose).

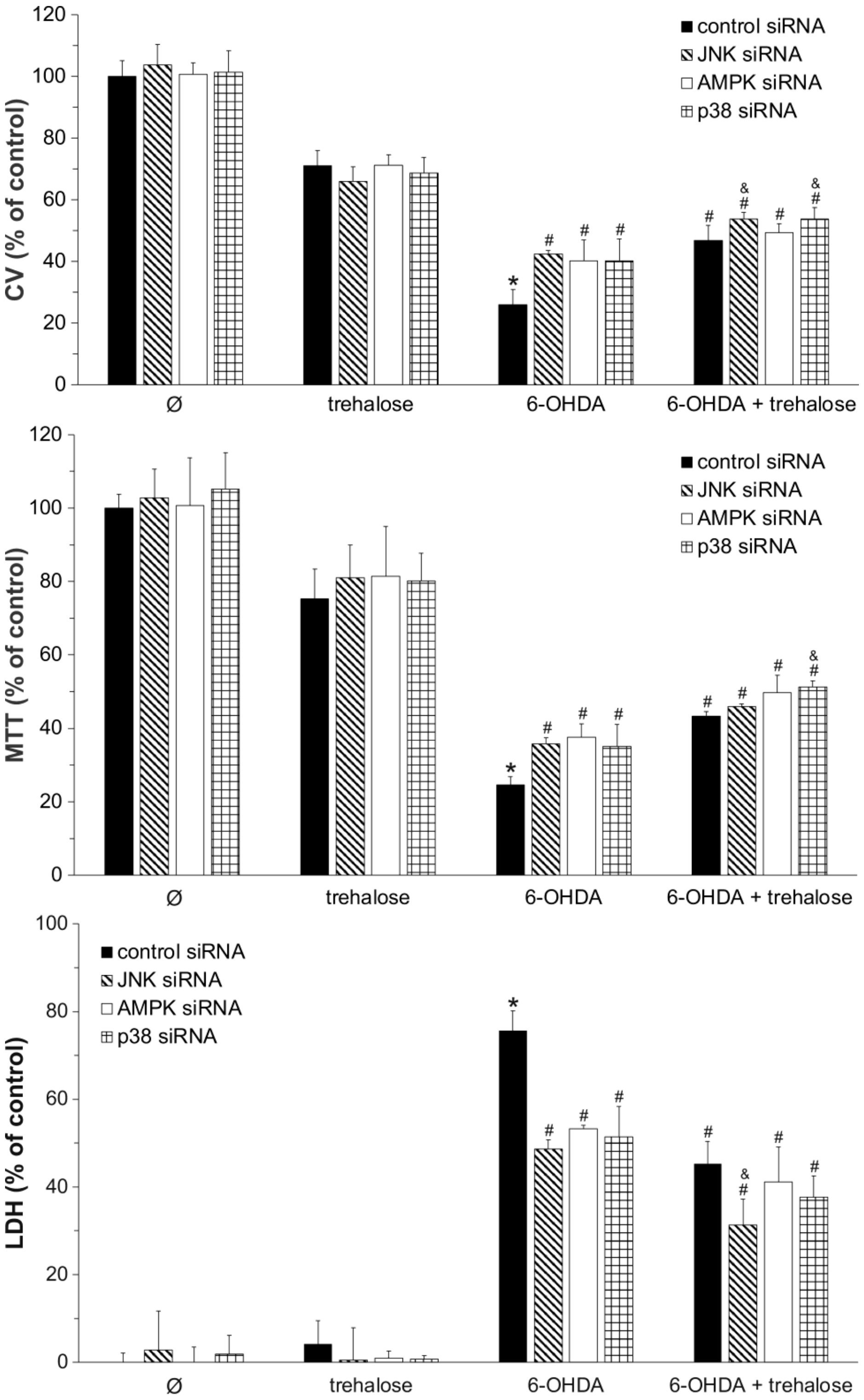

Figure S4. Inhibition of JNK, AMPK, and p38 MAPK is involved in trehalose-mediated protection from 6-OHDA. After transfection with control siRNA or siRNA against JNK, AMPK, or p38 MAPK, SH-SY5Y cells were incubated with or without trehalose (100 mM) for 24 h and then treated or not with 6-OHDA (60 µM) for another 24 h. Cell numbers were determined by crystal violet (CV) and MTT assays, and the data are presented as mean ± SD values of triplicates from a representative of three independent experiments (*$p < 0.05$ vs. control siRNA/no treatment; #$p < 0.05$ vs. control siRNA/6-OHDA; $^{\&}p < 0.05$ vs. control siRNA/6-OHDA + trehalose).

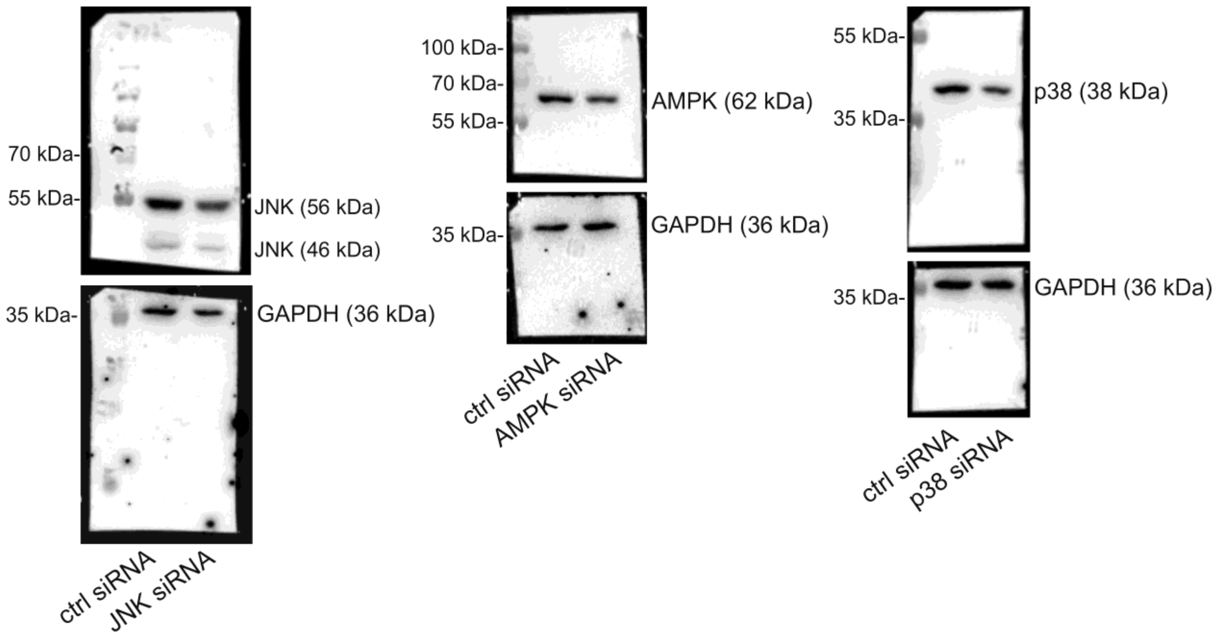

Figure S5. Original immunoblots confirming JNK, AMPK, and p38 MAPK downregulation by siRNA.
